# Supplementary material for: Hybrid Models and Biological Model Reduction with PyDSTool
Source: PLoS Comput Biol. 2012 Aug 9;8(8):e1002628. doi: 10.1371/journal.pcbi.1002628 (PMC3415397; doi:10.1371/journal.pcbi.1002628)
Supplement: Text S4 — Complete source code for the PyDSTool package (version 0.88.120504). Includes API documentation and help files linking to web pages. This file is identical to the current public release on Sourceforge.net. (ZIP) [file pcbi.1002628.s004.zip › PyDSTool/html/PyDSTool.Toolbox.fracdim-module.html]

xml version="1.0" encoding="ascii"?


PyDSTool.Toolbox.fracdim


| Home | Trees | Indices | Help | PyDSTool |
| --- | --- | --- | --- | --- |

Package PyDSTool ::
Package Toolbox ::
Module fracdim

[hide private]

[frames] | no frames]

# Module fracdim

source code

Fractal dimension estimates for analysis of datasets having rank 2 or
3.

A journal publication concerning the use of these functions appears in
IEEE Transactions on BioMedical Engineering, 2008.

(c) 2005, 2006. Robert Clewley, John Guckenheimer.


|  |  |
| --- | --- |
| Functions | [hide private] |

|  |  |
| --- | --- |
| corr\_dim(data, which\_norm=2)  Correlation dimension of a data set. | source code |

|  |  |
| --- | --- |
| corr\_dim\_with\_progress(data, inforate=10000, which\_norm=2)  Version of correlation dimension with text file progress dump, at a sample rate of 'inforate', because the calculation typically takes a long time. | source code |

|  |  |
| --- | --- |
| timeseq(time, covering, refpt)  Extract time sequence from a covering | source code |

|  |  |
| --- | --- |
| find\_cover(data, remain\_tol=5, step=None, initref=None, wlo=1, whi=1, check\_large\_nhds=True, large\_nhd=300, min\_nhd\_size=50, min\_ix=25, max\_res=1.0, max\_std=0.2, quiet=True)  OUTPUTS: | source code |

|  |  |
| --- | --- |
| find\_nhd(data, tol\_up=0.2, tol\_down=0.05, quiet=True, max\_res=0.2, max\_std=0.1, step=None, initref=None, min\_ix=10, min\_nhd\_size=20, max\_radius\_ratio=1.5)  Find a single neighbourhood around a reference point. | source code |

|  |  |
| --- | --- |
| do\_stats(covering, covered, maxD, bin\_width=1, fignum=1, num\_panels=4, s\_frac=0.5, nhd\_size\_thresh=None, nhd\_max\_plot\_size=None, minD=None, D\_estimate\_method=`'``absolute``'`, weight\_by\_size=False, force\_xaxis\_limits=False, radius\_yaxis\_limits=None, save=`'``'`)  Produces statistics of pointwise dimension estimates of a data set, including a histogram of the distribution of neighbourhood dimensions. | source code |

|  |  |
| --- | --- |
| get\_filtered\_ixs(cover\_by\_dimix)  Returns the list of indices in covering for which the neighbourhood size was larger than the threshold for dimension binning. | source code |

|  |  |
| --- | --- |
| find\_outliers(data, plotdata, cluster\_dim, cluster\_width, max\_central=50) | source code |

|  |  |
| --- | --- |
| get\_cover\_radii(covering, ixs=None, method=`'``divide``'`)  Return the radius of a covering | source code |

|  |  |
| --- | --- |
| filter\_by\_radius(covering, radii, rlo=None, rhi=None)  Returns list of filtered (index, radius) pairs and corresponding sub-coverings' dimensions | source code |

|  |  |
| --- | --- |
| plot\_radius\_distribution(radii, max\_radius, radius\_bin\_width)  Expects radii dictionary, as returned by get\_cover\_radii. | source code |

|  |  |
| --- | --- |
| plot\_dim\_by\_thickness(covering, radii, rwidth=0, plotstyle=`'``k.``'`)  Plot of dimensions of neighbourhoods with thickness r, using binned data of bin width rwidth. | source code |

|  |  |
| --- | --- |
| plot\_thickness\_by\_dim(covering, radii, plotstyle=`'``k.``'`)  Plot of thickness of neighbourhoods as a function of their estimated dimension. | source code |

|  |  |
| --- | --- |
| nhd(data, covering, refpt) | source code |

|  |  |
| --- | --- |
| get\_rV\_curves(data, refpts, doplot=True, show\_seq=False)  Get r-V curves for pointwise dimension estimate of data | source code |

|  |  |
| --- | --- |
| make\_secant\_fig\_dict(items) | source code |

|  |  |
| --- | --- |
| prep\_secant\_figure(data, plotdata, ssorted, radii, ixdata, sstats, start\_ix, stop\_ix, Delta, fignum, save=`'``'`, show\_seq=False) | source code |

|  |  |
| --- | --- |
| slope\_range(data, refptix, step=2, Delta=10, startix=10, stopix=None, doplot=False, maxD=inf)  Slope range by secants over a range of log(Delta) ball volumes | source code |

|  |  |
| --- | --- |
| scatterplot\_slopes(data, num\_samples=None, startix=10, stopix=None, Delta=10, marker=`'``o``'`, marker\_size=40, cmap=cm.hot, color\_source=None, step=5, fignum=None, maxD=inf)  fignum = 0 switches off plotting (just return statistics) | source code |

|  |  |
| --- | --- |
| sorted\_by\_slope(plotdata, select=0)  select: 0 = rmin, 1 = rmax | source code |

|  |  |
| --- | --- |
| sorted\_by\_radius(colors, select=0)  select: 0 = rmin, 1 = rmax | source code |

|  |  |
| --- | --- |
| find\_from\_sorted(x, v, next\_largest=1, indices=None) | source code |

|  |  |
| --- | --- |
| rescatter(plotdata, colors, color\_source=None, marker=`'``o``'`, marker\_size=40, cmap=cm.hot, newfigure=True) | source code |

|  |  |
| --- | --- |
| scatter\_histo(plotdata, select=1, bin\_width=1, maxD=None, xlim=None, ylim=None) | source code |

|  |  |
| --- | --- |
| PD\_E(a, secfig=5, verbose=False, saveplot=True, force=False)  Pointwise dimension estimation (PD-E) using secants of r-V curves. | source code |


|  |  |
| --- | --- |
| Variables | [hide private] |

ALLOW\_THREADS = `1`

Abs = `Abs (ModelSpec wrapper)`

Acos = `Acos (ModelSpec wrapper)`

Asin = `Asin (ModelSpec wrapper)`

Atan = `Atan (ModelSpec wrapper)`

Atan2 = `Atan2 (ModelSpec wrapper)`

BUFSIZE = `10000`

Betavariate = `Betavariate (ModelSpec wrapper)`

CLIP = `0`

Ceil = `Ceil (ModelSpec wrapper)`

Choice = `Choice (ModelSpec wrapper)`

Cos = `Cos (ModelSpec wrapper)`

Cosh = `Cosh (ModelSpec wrapper)`

Degrees = `Degrees (ModelSpec wrapper)`

E = `QuantSpec e (ExpFuncSpec)`

ERR\_CALL = `3`

ERR\_DEFAULT = `0`

ERR\_DEFAULT2 = `2084`

ERR\_IGNORE = `0`

ERR\_LOG = `5`

ERR\_PRINT = `4`

ERR\_RAISE = `2`

ERR\_WARN = `1`

Exp = `Exp (ModelSpec wrapper)`

Expovariate = `Expovariate (ModelSpec wrapper)`

FLOATING\_POINT\_SUPPORT = `1`

FPE\_DIVIDEBYZERO = `1`

FPE\_INVALID = `8`

FPE\_OVERFLOW = `2`

FPE\_UNDERFLOW = `4`

Fabs = `Fabs (ModelSpec wrapper)`

False\_ = `False`

Floor = `Floor (ModelSpec wrapper)`

Fmod = `Fmod (ModelSpec wrapper)`

Frexp = `Frexp (ModelSpec wrapper)`

Gammavariate = `Gammavariate (ModelSpec wrapper)`

Gauss = `Gauss (ModelSpec wrapper)`

Getrandbits = `Getrandbits (ModelSpec wrapper)`

Getstate = `Getstate (ModelSpec wrapper)`

Hypot = `Hypot (ModelSpec wrapper)`

Infinity = `inf`

Jumpahead = `Jumpahead (ModelSpec wrapper)`

Ldexp = `Ldexp (ModelSpec wrapper)`

Log = `Log (ModelSpec wrapper)`

Log10 = `Log10 (ModelSpec wrapper)`

Lognormvariate = `Lognormvariate (ModelSpec wrapper)`

MAXDIMS = `32`

Max = `Max (ModelSpec wrapper)`

Min = `Min (ModelSpec wrapper)`

Modf = `Modf (ModelSpec wrapper)`

NAN = `nan`

NINF = `-inf`

NZERO = `-0.0`

Normalvariate = `Normalvariate (ModelSpec wrapper)`

PINF = `inf`

PZERO = `0.0`

Paretovariate = `Paretovariate (ModelSpec wrapper)`

Pi = `QuantSpec pi (ExpFuncSpec)`

Pow = `Pow (ModelSpec wrapper)`

RAISE = `2`

Radians = `Radians (ModelSpec wrapper)`

Randint = `Randint (ModelSpec wrapper)`

Random = `Random (ModelSpec wrapper)`

Randrange = `Randrange (ModelSpec wrapper)`

SHIFT\_DIVIDEBYZERO = `0`

SHIFT\_INVALID = `9`

SHIFT\_OVERFLOW = `3`

SHIFT\_UNDERFLOW = `6`

Sample = `Sample (ModelSpec wrapper)`

ScalarType = `(<type 'int'>, <type 'float'>, <type 'complex'>, ...`

Seed = `Seed (ModelSpec wrapper)`

Setstate = `Setstate (ModelSpec wrapper)`

Shuffle = `Shuffle (ModelSpec wrapper)`

Sin = `Sin (ModelSpec wrapper)`

Sinh = `Sinh (ModelSpec wrapper)`

Sqrt = `Sqrt (ModelSpec wrapper)`

Sum = `Sum (ModelSpec wrapper)`

Systemrandom = `Systemrandom (ModelSpec wrapper)`

Tan = `Tan (ModelSpec wrapper)`

Tanh = `Tanh (ModelSpec wrapper)`

True\_ = `True`

UFUNC\_BUFSIZE\_DEFAULT = `10000`

UFUNC\_PYVALS\_NAME = `'UFUNC_PYVALS'`

Uniform = `Uniform (ModelSpec wrapper)`

Vonmisesvariate = `Vonmisesvariate (ModelSpec wrapper)`

WRAP = `1`

Weibullvariate = `Weibullvariate (ModelSpec wrapper)`

Wichmannhill = `Wichmannhill (ModelSpec wrapper)`

absolute = `<ufunc 'absolute'>`

add = `<ufunc 'add'>`

bitwise\_and = `<ufunc 'bitwise_and'>`

bitwise\_not = `<ufunc 'invert'>`

bitwise\_or = `<ufunc 'bitwise_or'>`

bitwise\_xor = `<ufunc 'bitwise_xor'>`

c\_ = `<numpy.lib.index_tricks.CClass object at 0x1154330>`

cast = `{<type 'numpy.int64'>: <function <lambda> at 0x10a8bb0>...`

conj = `<ufunc 'conjugate'>`

conjugate = `<ufunc 'conjugate'>`

copysign = `<ufunc 'copysign'>`

deg2rad = `<ufunc 'deg2rad'>`

divide = `<ufunc 'divide'>`

equal = `<ufunc 'equal'>`

exp2 = `<ufunc 'exp2'>`

expm1 = `<ufunc 'expm1'>`

floor\_divide = `<ufunc 'floor_divide'>`

fmax = `<ufunc 'fmax'>`

fmin = `<ufunc 'fmin'>`

greater\_equal = `<ufunc 'greater_equal'>`

index\_exp = `<numpy.lib.index_tricks.IndexExpression object at ...`

inf = `inf`

infty = `inf`

invert = `<ufunc 'invert'>`

isinf = `<ufunc 'isinf'>`

left\_shift = `<ufunc 'left_shift'>`

little\_endian = `True`

log1p = `<ufunc 'log1p'>`

logaddexp = `<ufunc 'logaddexp'>`

logaddexp2 = `<ufunc 'logaddexp2'>`

logical\_and = `<ufunc 'logical_and'>`

logical\_not = `<ufunc 'logical_not'>`

logical\_xor = `<ufunc 'logical_xor'>`

maximum = `<ufunc 'maximum'>`

mgrid = `<numpy.lib.index_tricks.nd_grid object at 0x113d490>`

minimum = `<ufunc 'minimum'>`

multiply = `<ufunc 'multiply'>`

n = `9`

nan = `nan`

nbytes = `{<type 'numpy.int64'>: 8, <type 'numpy.int16'>: 2, <t...`

negative = `<ufunc 'negative'>`

nextafter = `<ufunc 'nextafter'>`

not\_equal = `<ufunc 'not_equal'>`

ogrid = `<numpy.lib.index_tricks.nd_grid object at 0x113dbb0>`

ones\_like = `<ufunc 'ones_like'>`

rad2deg = `<ufunc 'rad2deg'>`

reciprocal = `<ufunc 'reciprocal'>`

remainder = `<ufunc 'remainder'>`

right\_shift = `<ufunc 'right_shift'>`

rint = `<ufunc 'rint'>`

s\_ = `<numpy.lib.index_tricks.IndexExpression object at 0x11543f0>`

sctypeDict = `{0: <type 'numpy.bool_'>, 1: <type 'numpy.int8'>,...`

sctypeNA = `{'?': 'Bool', 'B': 'UInt8', 'Bool': <type 'numpy.bo...`

sctypes = `{'complex': [<type 'numpy.complex64'>, <type 'numpy....`

signbit = `<ufunc 'signbit'>`

spacing = `<ufunc 'spacing'>`

square = `<ufunc 'square'>`

subtract = `<ufunc 'subtract'>`

t = `'0'`

true\_divide = `<ufunc 'true_divide'>`

trunc = `<ufunc 'trunc'>`

typeDict = `{0: <type 'numpy.bool_'>, 1: <type 'numpy.int8'>, 2...`

typeNA = `{'?': 'Bool', 'B': 'UInt8', 'Bool': <type 'numpy.bool...`

typecodes = `{'All': '?bhilqpBHILQPfdgFDGSUVOMm', 'AllFloat': '...`


|  |  |
| --- | --- |
| Function Details | [hide private] |

|  |  |
| --- | --- |
| find\_cover(data, remain\_tol=5, step=None, initref=None, wlo=1, whi=1, check\_large\_nhds=True, large\_nhd=300, min\_nhd\_size=50, min\_ix=25, max\_res=1.0, max\_std=0.2, quiet=True) | source code |

```
OUTPUTS:

 covering is a dict:
    reference point -> (dim, low radius, high radius, covered_indices)
 covered is a dict:
    point -> reference points whose neighbourhoods include it
```

|  |  |
| --- | --- |
| do\_stats(covering, covered, maxD, bin\_width=1, fignum=1, num\_panels=4, s\_frac=0.5, nhd\_size\_thresh=None, nhd\_max\_plot\_size=None, minD=None, D\_estimate\_method=`'``absolute``'`, weight\_by\_size=False, force\_xaxis\_limits=False, radius\_yaxis\_limits=None, save=`'``'`) | source code |

```
Produces statistics of pointwise dimension estimates of a data set,
including a histogram of the distribution of neighbourhood dimensions.

R. Clewley, June 2006.

INPUTS:

maxD sets the maximum dimension to cater for.

bin_width selects the width of bins for dimensions (default 1).

fignum forces a particular figure number to be used for display.
    (Use fignum=0 to suppress plotting of the results and just return
     the statistics.)

num_panels argument must be 2 (for just nhd size and bin histo) or
   4 (also include min and max nhd radius).

s_frac selects the fraction (or multiple) of the standard deviation of
   neighbourhood sizes to use as the cutoff for the "bulk" of the data
   set, in order to avoid including large outliers in determining
   nhd_size_threshold (default 0.5).

nhd_size_threshold selects the smallest neighbourhood size to be
   included in histogram of neighbourhood dimensionalities
   (default None => automatic selection, i.e., the mean of the first
    s_frac of the neighbourhood sizes' standard deviation).
   Set to zero to prevent it being used or plotted.

nhd_max_plot_size selects the y-axis limit on the plot of neighbourhood
   sizes (default None => automatic selection).

D_estimate_method can be 'absolute' (uses cutoff of size = 1 to estimate D
   in histogram tail), or 'relative' (uses a cutoff = %age of peak binned
   neighbourhoods)

force_xaxis_limits (Boolean) determines whether x-axis limits should be
   forced to be the value of the (minD,maxD) parameters.

minD sets the minimum dimension to cater for (default to auto-choice).

radius_yaxis_limits (pair) sets the r_min and r_max y-axis upper limits,
   respectively.

save (string) sets a filename to use for saving the figure in available
   formats (so don't include a file extension).


OUTPUTS:

d_stats: dictionary {"D_tail": estimated D from tail,
                     "D_mean": histo mean,
                     "D_std": histo std dev}

dbins: Pointset of D bins

cover_by_dimix: dictionary of covers by index of dimensions binned
   (i.e., [0, bin_width, 2*bin_width, ..., maxD+1])

cover_by_size: list of neighbourhood coverings ordered by their size

cbu: array indicating how many points in overlap between neighbourhoods
    (array index corresponds to a number of points shared,
     value = number of nhds with that number of points shared)

ixs: list of indices of neighbourhoods of size > nhd_size_threshold

(integral: internal diagnostic feature)
```

|  |  |
| --- | --- |
| plot\_radius\_distribution(radii, max\_radius, radius\_bin\_width) | source code |

Expects radii dictionary, as returned by get\_cover\_radii. Returns
figure handle.

|  |  |
| --- | --- |
| plot\_dim\_by\_thickness(covering, radii, rwidth=0, plotstyle=`'``k.``'`) | source code |

Plot of dimensions of neighbourhoods with thickness r, using binned
data of bin width rwidth. r is defined as the neighbourhood's r\_max -
r\_min.

If rwidth=0 (default) then no data binning is done. Returns figure
handle.

|  |  |
| --- | --- |
| plot\_thickness\_by\_dim(covering, radii, plotstyle=`'``k.``'`) | source code |

Plot of thickness of neighbourhoods as a function of their estimated
dimension. r is defined as the neighbourhood's r\_max - r\_min.

Returns figure handle.

|  |  |
| --- | --- |
| PD\_E(a, secfig=5, verbose=False, saveplot=True, force=False) | source code |

Pointwise dimension estimation (PD-E) using secants of r-V curves.
See:

J. Guckenheimer, "Dimension Estimates for Attractors",
Contemporary Mathematics, Vol. 28, 1984.

R. Clewley, J. Guckenheimer, F. Valero-Cuevas, "Estimating
effective degrees of freedom in motor control", IEEE Transactions in
Biomedical Engineering, 2007.

  


|  |  |
| --- | --- |
| Variables Details | [hide private] |

### ScalarType

Value:
:   ```
    (<type 'int'>,
     <type 'float'>,
     <type 'complex'>,
     <type 'long'>,
     <type 'bool'>,
     <type 'str'>,
     <type 'unicode'>,
     <type 'buffer'>,
    ...
    ```

### cast

Value:
:   ```
    {<type 'numpy.int64'>: <function <lambda> at 0x10a8bb0>, <type 'numpy.
    int16'>: <function <lambda> at 0x10a8bf0>, <type 'numpy.complex128'>: 
    <function <lambda> at 0x10a8c30>, <type 'numpy.uint64'>: <function <la
    mbda> at 0x10a8c70>, <type 'numpy.complex256'>: <function <lambda> at 
    0x10a8cf0>, <type 'numpy.float32'>: <function <lambda> at 0x10a8d30>, 
    <type 'numpy.bool_'>: <function <lambda> at 0x10a8cb0>, <type 'numpy.u
    int8'>: <function <lambda> at 0x10a8db0>, <type 'numpy.int32'>: <funct
    ion <lambda> at 0x10a8eb0>, <type 'numpy.int8'>: <function <lambda> at
    ...
    ```

### index\_exp

Value:
:   ```
    <numpy.lib.index_tricks.IndexExpression object at 0x11543b0>
    ```

### nbytes

Value:
:   ```
    {<type 'numpy.int64'>: 8, <type 'numpy.int16'>: 2, <type 'numpy.comple
    x128'>: 16, <type 'numpy.uint64'>: 8, <type 'numpy.bool_'>: 1, <type '
    numpy.complex256'>: 32, <type 'numpy.float32'>: 4, <type 'numpy.int8'>
    : 1, <type 'numpy.uint8'>: 1, <type 'numpy.uint16'>: 2, <type 'numpy.o
    bject_'>: 4, <type 'numpy.float64'>: 8, <type 'numpy.int32'>: 4, <type
     'numpy.string_'>: 0, <type 'numpy.void'>: 0, <type 'numpy.float128'>:
     16, <type 'numpy.int32'>: 4, <type 'numpy.uint32'>: 4, <type 'numpy.u
    nicode_'>: 0, <type 'numpy.complex64'>: 8, <type 'numpy.uint32'>: 4}
    ```

### sctypeDict

Value:
:   ```
    {0: <type 'numpy.bool_'>,
     1: <type 'numpy.int8'>,
     2: <type 'numpy.uint8'>,
     3: <type 'numpy.int16'>,
     4: <type 'numpy.uint16'>,
     5: <type 'numpy.int32'>,
     6: <type 'numpy.uint32'>,
     7: <type 'numpy.int32'>,
    ...
    ```

### sctypeNA

Value:
:   ```
    {'?': 'Bool',
     'B': 'UInt8',
     'Bool': <type 'numpy.bool_'>,
     'Complex128': <type 'numpy.complex256'>,
     'Complex32': <type 'numpy.complex64'>,
     'Complex64': <type 'numpy.complex128'>,
     'D': 'Complex64',
     'F': 'Complex32',
    ...
    ```

### sctypes

Value:
:   ```
    {'complex': [<type 'numpy.complex64'>,
                 <type 'numpy.complex128'>,
                 <type 'numpy.complex256'>],
     'float': [<type 'numpy.float32'>,
               <type 'numpy.float64'>,
               <type 'numpy.float128'>],
     'int': [<type 'numpy.int8'>,
             <type 'numpy.int16'>,
    ...
    ```

### typeDict

Value:
:   ```
    {0: <type 'numpy.bool_'>,
     1: <type 'numpy.int8'>,
     2: <type 'numpy.uint8'>,
     3: <type 'numpy.int16'>,
     4: <type 'numpy.uint16'>,
     5: <type 'numpy.int32'>,
     6: <type 'numpy.uint32'>,
     7: <type 'numpy.int32'>,
    ...
    ```

### typeNA

Value:
:   ```
    {'?': 'Bool',
     'B': 'UInt8',
     'Bool': <type 'numpy.bool_'>,
     'Complex128': <type 'numpy.complex256'>,
     'Complex32': <type 'numpy.complex64'>,
     'Complex64': <type 'numpy.complex128'>,
     'D': 'Complex64',
     'F': 'Complex32',
    ...
    ```

### typecodes

Value:
:   ```
    {'All': '?bhilqpBHILQPfdgFDGSUVOMm',
     'AllFloat': 'fdgFDG',
     'AllInteger': 'bBhHiIlLqQpP',
     'Character': 'c',
     'Complex': 'FDG',
     'Datetime': 'Mm',
     'Float': 'fdg',
     'Integer': 'bhilqp',
    ...
    ```

  


| Home | Trees | Indices | Help | PyDSTool |
| --- | --- | --- | --- | --- |

|  |  |
| --- | --- |
| Generated by Epydoc 3.0.1 on Fri May 4 15:24:04 2012 | http://epydoc.sourceforge.net |
